# An Overview on Personal Protective Equipment (PPE) Fabricated with Additive Manufacturing Technologies in the Era of COVID-19 Pandemic

**DOI:** 10.3390/polym12112703

**Published:** 2020-11-16

**Authors:** Szilard Rendeki, Balint Nagy, Matyas Bene, Attila Pentek, Luca Toth, Zalan Szanto, Roland Told, Peter Maroti

**Affiliations:** 1 Medical Simulation Education Center, Medical School, University of Pecs, 7624 Pecs, Hungary; rendeki.szilard@pte.hu; 2 Department of Operational Medicine, Medical School, University of Pecs, 7624 Pecs, Hungary; balintjanosnagy@gmail.com; 3 Department of Anaesthesiology and Intensive Therapy, Medical School, University of Pecs, 7624 Pecs, Hungary; 4 3D Printing and Visualization Centre, University of Pecs, 7624 Pecs, Hungary; bene.matyas@pte.hu (M.B.); pentek.attila@pte.hu (A.P.); tothluca.pte@gmail.com (L.T.); told.roland@pte.hu (R.T.); 5 Department of Neurosurgery, Medical School, University of Pecs, 7623 Pecs, Hungary; 6 Institute for Translational Medicine, Medical School, University of Pecs, 7624 Pecs, Hungary; 7 Department of Surgery, Medical School, University of Pecs, 7624 Pecs, Hungary; szantozalan@gmail.com

**Keywords:** additive manufacturing, 3D printing, polymers, PLA, polyamide, personal protective equipment, remote medicine, sterilization, COVID-19, mechanical testing

## Abstract

Different additive manufacturing technologies have proven effective and useful in remote medicine and emergency or disaster situations. The coronavirus disease 2019 (COVID-19) disease, caused by the Severe Acute Respiratory Syndrome Coronavirus 2 (SARS-CoV-2) virus, has had a huge impact on our society, including in relation to the continuous supply of personal protective equipment (PPE). The aim of the study is to give a detailed overview of 3D-printed PPE devices and provide practical information regarding the manufacturing and further design process, as well as describing the potential risks of using them. Open-source models of a half-face mask, safety goggles, and a face-protecting shield are evaluated, considering production time, material usage, and cost. Estimations have been performed with fused filament fabrication (FFF) and selective laser sintering (SLS) technology, highlighting the material characteristics of polylactic acid (PLA), polyamide, and a two-compound silicone. Spectrophotometry measurements of transparent PMMA samples were performed to determine their functionality as goggles or face mask parts. All the tests were carried out before and after the tetra-acetyl-ethylene-diamine (TAED)-based disinfection process. The results show that the disinfection has no significant effect on the mechanical and structural stability of the used polymers; therefore, 3D-printed PPE is reusable. For each device, recommendations and possible means of development are explained. The files of the modified models are provided. SLS and FFF additive manufacturing technology can be useful tools in PPE development and small-series production, but open-source models must be used with special care.

## 1. Introduction

Additive manufacturing (AM) technologies have reshaped medical diagnostics, preventive and preoperative measures, treatment and rehabilitation, as have tissue engineering processes in recent years [1,2,3,4,5,6,7,8,9,10,11,12,13,14,15]. Previous studies have shown that 3D printing in medicine is mainly used due to the possibility of easy customization and cost-effectiveness; additionally, the majority of applications are based on material extrusion technologies using polymers [13,14,15,16]. The most common type of 3D printing technology is fused filament fabrication (FFF); therefore, these products are prevalent worldwide [13,15]. Selective laser sintering (SLS) technology is prominent and accessible in medical 3D printing, for which the most frequently used materials are polyamide (PA) and polyether ether ketone (PEEK) [17,18,19,20]. Furthermore, 3D printing has a significant role in remote medicine when the supplies and availability of resources are greatly limited, e.g., in space missions, in remote regions, and during emergency or disaster situations [21,22,23,24,25,26].

In the last month of 2019, a new disease battered the people of Wuhan City, Hubei, China [27,28]. The novel coronavirus, named Severe Acute Respiratory Syndrome Coronavirus 2 (SARS-CoV-2), led to an epidemic in China and expanded into an ongoing global pandemic, infecting more than 60,000 people daily by the end of March 2020, and overall, cca. 42 million cases worldwide and 1.1 million deaths were reported from all continents except Antarctica [29,30,31,32]. Compared to other coronaviruses such as SARS-CoV or Middle East respiratory syndrome coronavirus (MERS), the overall mortality of SARS-CoV-2 is lower; however, the relative infection incidence is over 10 times greater. Therefore, the World Health Organization (WHO) declared a Public Health Emergency of International Concern in January 2020 [31,32]. Considering that there is no definitive treatment available to treat COVID-19 patients, the prevention of transmission is extremely important [31,32,33]. The viral transmission possibly occurs from asymptomatic or mildly ill patients; therefore, the WHO and the Centers for Disease Control and Prevention (CDC) emphasized the importance of preventive measures, including social distancing and the proper use of personal protective equipment (PPE) [33,34,35]. Based on the current recommendations of the CDC and WHO, people should wear non-medical face masks in public areas, public transport, meeting and events, generally anywhere around other people, in order to prevent the spread of the virus [34,35,36,37]. Wearing a medical facial mask is required for the patients who have the typical symptoms of COVID infection, such as coughing, fever, or gastrointestinal complaints and is beneficial in avoiding the spread of the virus, especially for people at high risk, such as those over 60 years old or with comorbidities such as chronic obstructive pulmonary disease [36,37]. Furthermore, the use of face masks is essential for medical and healthcare personnel during clinical practice or care of patients suffering from COVID-19 [33,35,36].

COVID-19 is transmitted primarily by respiratory droplets, close physical contact, and airborne transmission to a smaller extent. Consequently, frequent disinfection is essential to prevent transmission [31,35,37,38]. SARS-CoV-2 is an RNA virus with a lipid bilayer (envelope), which makes it more sensitive to disinfectants. Thus, it belongs to the least-resistant pathogens on the standardized resistance scale against germicidal chemicals [16,31,32,38]. According to recent data, many different types of products and approaches are sufficient for medical and non-medical disinfection, such as chlorine-based products that generate hypochlorite (HOCl), 70–90% ethanol, hydrogen peroxide >0.5%, formaldehyde and glutaraldehyde, high temperature (75 °C for 30 min, 67 °C for 67 min), and ultraviolet light for 60 min [38,39,40,41,42,43,44]. Furthermore, the list of effective disinfectants against the COVID-19 virus has been released by the United States Environmental Protection Agency [45,46].

Compared to conventional technologies, the use of additive manufacturing in the medical field has several benefits, such as inner specific design, personalized layout, sustainability, and time reduction, in addition to minimalized transportation costs. It also has disadvantages, such as essential post-processing, limited available materials, skilled operator, and low production volume [14,15,16]. Based on the sudden supply-demand imbalance for PPE caused by the coronavirus disease 2019 (COVID-19), a large number of crafters, hobbyists, and even companies started to design and make 3D-printed PPE such as masks, shields, door openers, mask adjusters, and even respirator parts [15,16,32,33,44,47,48]. In the media, on different video-sharing platforms and social media sites, a tremendous number of publications, articles, and videos have shared how to produce masks, face shields, or goggles intended for civilian or professional healthcare-related use to improve the supply chain system [48]. This civilian-based initiative is honorable and important. However, critical evaluation of these devices is essential to provide detailed insight into these products and minimize potential risks and hazards in order to avoid the preventable loss of healthcare workers or patients due to the improper design or use of these devices [16]. Previous scientific publications are very limited regarding PPE fabricated by AM technologies. Using reverse engineering and AM methods, a 3D-printed face mask was created by SLS technology, and it was concluded that 3D printing can be a suitable tool for the further development and production of PPE [49,50]. Recent studies have also highlighted that additive manufacturing can be applied rapidly in case of supply shortage; however, further investigation is required regarding the designing protocol, suitable materials, sterilization, etc. [16,47,51]. Our aim is to provide an overview of the most common PPE, namely face shields, half masks, and goggles that are available on open source platforms, revealing the possible threats and hazards related to their medical use. Since most sanitizers have been tested on conventional devices and surfaces, our goal is to assess how disinfection affects the mechanical properties and flex resistance of the 3D-printed devices along with transparency analysis in case of vision and face protective appliances. Furthermore, development recommendations should be highlighted to reduce the production time and costs and possibly enhance the protection level. We share and distribute our design data for further work in the field to enhance and support valid clinical research projects based on our results.

## 2. Materials and Methods

### 2.1. 3D Printing Technology, Materials, and Disinfection Protocol

To evaluate the feasibility of different 3D printing technologies, specimens and model equipment were printed. Based on previous studies, it was observed that for medical device development and small-series production, the most frequently used technologies worldwide are FFF and SLS 3D printing. For the FFF 3D printing, a Craftunique Craftbot 2 Plus with Craftware™ slicing software and polylactic acid (PLA, distributed and manufactured by Herz Hungária Ltd., Ullo, Hungary) was used for the estimation and production of PPE. The devices and PLA test specimens were printed with a 0.6 mm nozzle diameter and 400 µm layer height for the face shields and a 0.4 mm nozzle diameter and 200 µm layer height for all other cases. The printing speed was set to 60 mm/s, and the infill density was 100%. For estimating the productivity and for manufacturing test bars made of PA (PA2200—Varinex Ltd., Budapest, Hungary), an EOS Formiga P110 was used. The layer height was set to 100 µm resolution. The printing speed was 5 sec/layer. The room temperature was set to 24 °C. According to international standards, 5 pieces of test bars were printed for each test. All of the test specimens were measured in the “X” printing orientation. In the development process of the face masks, a ZA-22 “THIXO BODY” (Alvin Ltd., Budakeszi, Hungary) two-compound silicone mixture was molded. For transparent parts of goggles and face shields, 0.3 mm thick PMMA (poly[methyl methacrylate]) sheets (distributed by Aka-Dekor Ltd., Pecs, Hungary) were applied. In order to assess the mechanical properties of the objects, based on international standards, all the material tests were performed on test specimens without disinfection, after 5 and 10 disinfection cycles, respectively. As a disinfection agent, the commonly used solution of tetra-acetyl-ethylene-diamine (TAED) and sodium-perborate solution (commercially available as Sekusept™—EcoLab Hungary Ltd., Budapest, Hungary) 2 *m*/*m*% solution was applied [40,45,46,52,53]. Based on the local and international medical protocol, one disinfection cycle was set for 1 h, and all the test specimens were submerged in the agent at room temperature (24 °C).

### 2.2. Mechanical and Structural Comparison of PLA, PA, and Silicone Materials

As a dynamic mechanical test, Charpy impact test (ISO 179-1) was used on the un-notched specimens with a size of 80 × 10 × 4 mm. For the static mechanical analysis, a 3-point bending test (ISO-178) and tensile strength test (ISO-527-2) were performed. The 3-point bending test was carried out on the specimens with a size of 80 × 10 × 4 mm, and tensile strength tests are specified as B1. A flex resistance test of the ZA-22 silicone materials was carried out according to the ISO 32,100 standard using a Zwick/Roell (Senselektro Ltd., Budapest, Hungary) e/m actuator. The size of the test specimen was 70 × 45 × 1 mm and the number of cycles was 1000, 2000, 3000, 4000, and 5000 in case of all disinfected and non-disinfected samples. Shore A hardness measurements were performed in the case of the silicone test specimens. All of the test bars were laid on the printing bed with the largest surface facing downwards. All of the test specimens were measured in the “X” printing orientation. The room temperature was set to 22.6 °C, while the relative humidity was 49.5%. According to international standards, 5 pieces of test bars were printed for each test. The broken surfaces of the test specimens were examined using scanning electron microscopy (SEM-JSM-6300, Jeol, Japan) at 10× and 60× magnification. Gold sheeting was applied to the test bars. After a flex resistance test, the surface of the silicone samples was examined with a König digital microscope with 55× magnification. For the statistical analysis, OriginPro 2018 software was used.

### 2.3. Spectrophotometry

To determine the effect of disinfection procedure on the transparency of PMMA sheets, spectrophotometry measurements were performed on test samples of 0.3 mm thick and 50 × 30 mm size samples using Secoman Anthelie Advanced 2 spectrophotometry device (Secomam, Ales, France). The change of intensity was measured, between 300 and 900 nm wavelength, on non-disinfected and disinfected test specimens. All measurements were repeated 5 times. 

### 2.4. Introduction of the Open-Source Models

**Figure 1:**
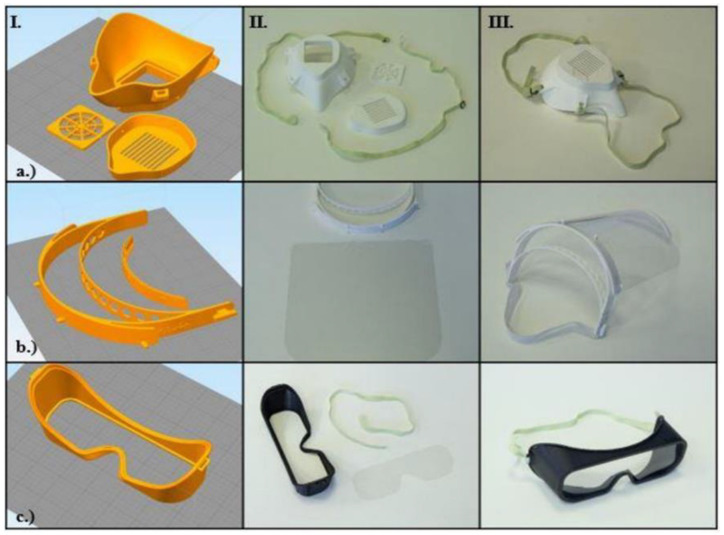
Overview of the open-source personal protective equipment (PPE) models. In the columns, the main steps of production and assembly are demonstrated (I—Craftware™ (Craftunique, 1087 Budapest, Hungary) software screenshot, before slicing, II—3D-printed models, III—assembled, finished models). The first row shows (**a**) the open-source (OS) half mask; the second row shows (**b**) the OS face shield; the third row shows (**c**) the OS safety goggles, according to the production steps.

According to publicly available materials and sources, three different types of PPE have been manufactured by 3D printing communities to date, mainly using FFF technology. Since biological agents transferred by droplets can easily enter the human body through mucous membranes, safety goggles can be used to specifically protect viral penetration via the eye. To protect the face from aerosol-based biological threats such as SARS-CoV-2, face masks can be used. This equipment is especially useful for all healthcare professionals who might come into contact with the virus. Protective half masks are important in filtering the air, which can reduce the risk of exposure to airborne diseases and aerosol-based microorganisms; therefore, they can be useful at all levels of the healthcare system. The effectiveness of half masks mainly relies on the type of incorporated filter and the fitting parameters to the face. Further protection can be achieved by applying face shields, either in combination with other PPEs or solely for non-medical purposes. Three main models of the aforementioned PPEs were examined, considering the production time, costs, and practical aspects. The following open-source (OS) models were examined: a half mask (“OS Half Mask” in the Supplementary Material; “NHS COVID MASK REMIX” by user wayneuk on Thingiverse), a face protection shield (“OS Shield” in the Supplementary Material; “PRUSA RC 2” by Prusa Research a.s.), and safety goggles (“OS Safety Goggles” in the Supplementary Material; “COVID-19 coronavirus goggle” by user jim0089 on Thingiverse) (Figure 1).

## 3. Results

### 3.1. Results of Mechanical and Structural Analyses before and after Disinfection

**Figure 2:**
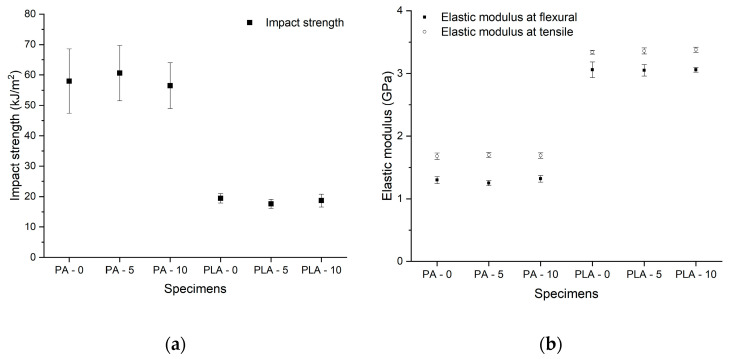
Results of mechanical tests. (**a**) The results of impact strength test (kJ/m^2^). The black squares indicate the means of the impact strength with the standard error in the case of the Charpy test. (**b**) The results of 3-point bending and tensile strength tests. The black squares indicate the means of elastic modulus with the standard error in the case of flexural test (GPa), while the dots show the means of elastic modulus in the case of tensile test (GPa), with the standard error. The test specimens are marked as: PA/PLA—0: Non-disinfected; PA/PLA—5: disinfected with 5 cycles; PA/PLA—10 disinfected with 10 cycles.

According to the data, PLA has significantly lower resistance against dynamic forces than PA (Figure 2). Before disinfection, the mean value of impact strength was 57.95 kJ/m^2^ ± 10.55 kJ/m^2^ for SLS test bars and 19.44 kJ/m^2^ ± 1.52 kJ/m^2^ for PLA test specimens. The results of the static mechanical test revealed that there is no significant difference in tensile strength: PLA had a mean value of 57.60 MP ± 1.22 MPa, while the mean value for PA test bars was defined as 46.40 MP ± 1.04 MPa. The three-point bending and tensile test showed significantly higher values for the FFF technology, with a remarkable elastic modulus of 3.06 GPa ± 0.12 GPa at three-point bending and 3.34 GPa ± 0.03 GPa at tensile test, while the SLS technology had mean values of 1.3 GPa ± 0.05 GPa at three-point bending and 1.68 GPa ± 0.05 GPa at tensile test. These results could be explained by the ISO-178-1 international standard, where the measurement ends at a 10% deflection. Neither the PA nor the PLA test bars broke at this rate, which implies a higher elasticity; therefore, the value of flexural stress at the standard deflection value could be determined more precisely. For PPE, dynamic forces are more relevant than other forces, since, during their everyday use, the PPE can be easily dropped or bumped into other objects; therefore, they have to be more resistant than other materials against these kinds of effects.

**Figure 3:**
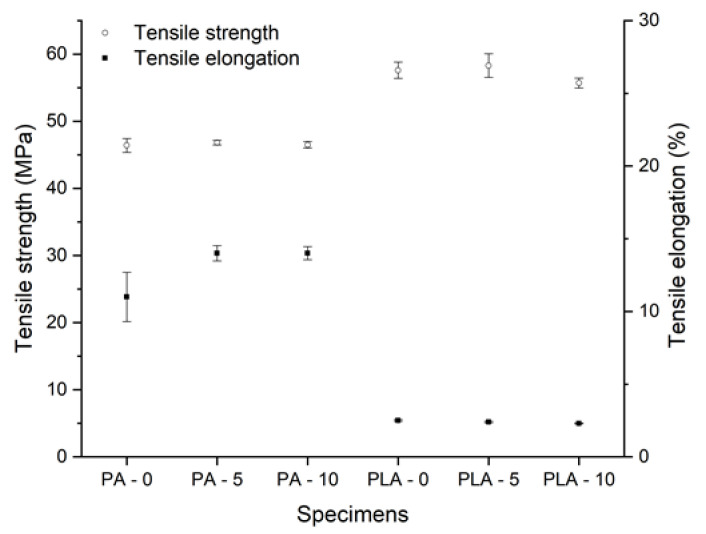
Results of tensile strength tests. The black squares indicate the means on tensile elongation (%) with the standard error, and the white dots show the mean values of tensile strength (MPa) with standard error. The test specimens are marked as: PA/PLA—0: Non-disinfected, PA/PLA—5: disinfected with 5 cycles, PA/PLA—10 disinfected with 10 cycles.

After submerging the test specimens in the Sekusept solution for 5 and 10 disinfection cycles, according to the data, no significant change was observed in the mechanical parameters. More surprisingly, in the case of polyamide, a small elevation of the elastic modulus was observed with the mean value of 1.32 GPa ± 0.05 GPa, as well as in the case of PLA with the mean value of 3.06 GPa ± 0.04 GPa (Figure 2b). The tensile elongation of PA also showed a slight increase, with the maximum value of 14% ± 0.45% (Figure 3).

**Figure 4:**
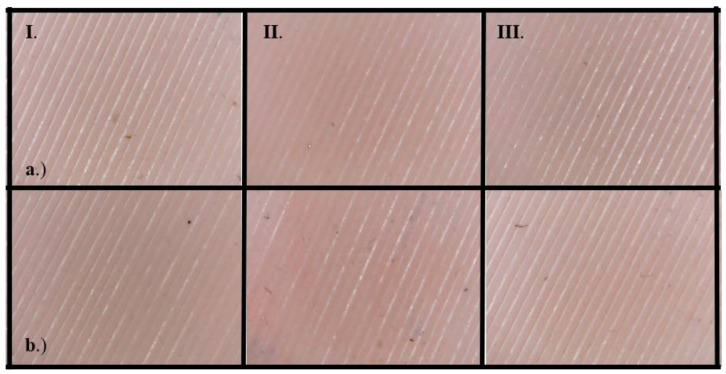
Results of flex resistance test of ZA-22 silicone. The numbers of the column refer to the number of disinfection cycles (I: non-disinfected, II: 5 cycles of disinfection, III: 10 cycles of disinfection). Row (**a**) marks the test specimens after 1000 flex test cycles; row (**b**) marks the specimens after 5000 flex cycles.

To determine the usability of ZA-22 silicone mold as an insulating layer in PPE, flex resistance tests and Shore A hardness tests were performed. As an interesting result, the surface analysis of the samples showed 0 (no change) degree of change in all cases, before and after the disinfection procedure as well (Figure 4). There was no significant change in Shore A hardness test on samples with and without the disinfection procedure, and the values varied between 18.92 ± 0.18 and 20.4 ± 0.58.

**Figure 5:**
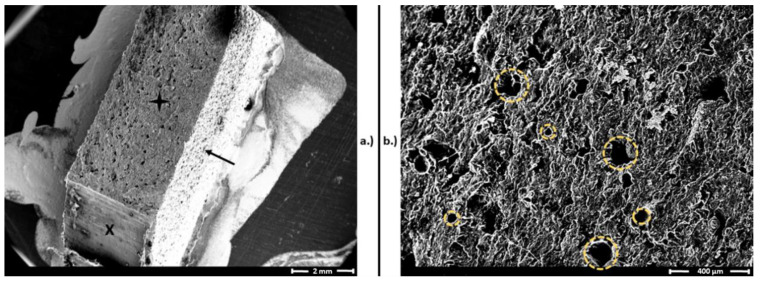
Scanning electron microscopy (SEM) images of a polyamide (PA) (selective laser sintering (SLS)) test bar after the Charpy impact test. (**a**) Broken surface of the test specimen, marked with a black star, similar to the intact surface (black arrow). The “X” represents a surface after cutting the test specimen to prepare the SEM sample. Magnification: 10×. (**b**) Surface and pores of PA test bars from the broken surface. The yellow dashed circles represent the pores, with sizes varying from 15 to 150 µm. Magnification: 60×.

Although there are many research papers describing the layer-based structural characteristics of FFF 3D printing technology, the examination of PA structures created with SLS technology focuses on the granule itself and the in-production states of melting [17,54,55,56,57]. In the case of PPE fabrication, the surface of the product is crucial for determining the survivability of different biological agents. According to the available evidence, the transmission of SARS-CoV-2 between people takes place via respiratory droplets with sizes >5–10 μm, while the size of the virus is approximately 120 nm [31,58,59,60]. Airborne transmission refers to the conveyance of pathogens via droplet nuclei, which have a diameter below 5 μm and spread over 1 m in air. SARS-CoV-2 transmission conceivably takes place under special circumstances such as during aerosol-generating medical procedures [59]. Therefore, the 15–150 μm pores of the SLS products without further coating, impregnation, lamination or other surface treatment method can serve as a place where SARS-CoV-2 can survive (Figure 5) without proper disinfection measures, and clinically used PPE tends to be highly contaminated with pathogens [13,47,61,62]. However, the virulent SARS-CoV-2 titer significantly decreases hourly, and decontamination by standard disinfection techniques such as sodium hypochlorite or detergents further decreases the possibility of viral spread and could be a potential solution to prevent contagion by PPE [27,62]. Since there is no effective treatment or vaccination against COVID-19, disease prevention is the highest priority; therefore, the standard disinfection methods should be further investigated and risk-stratified in terms of the aforementioned materials and reusable PPE.

### 3.2. Results of Spectrophotometry

**Figure 6:**
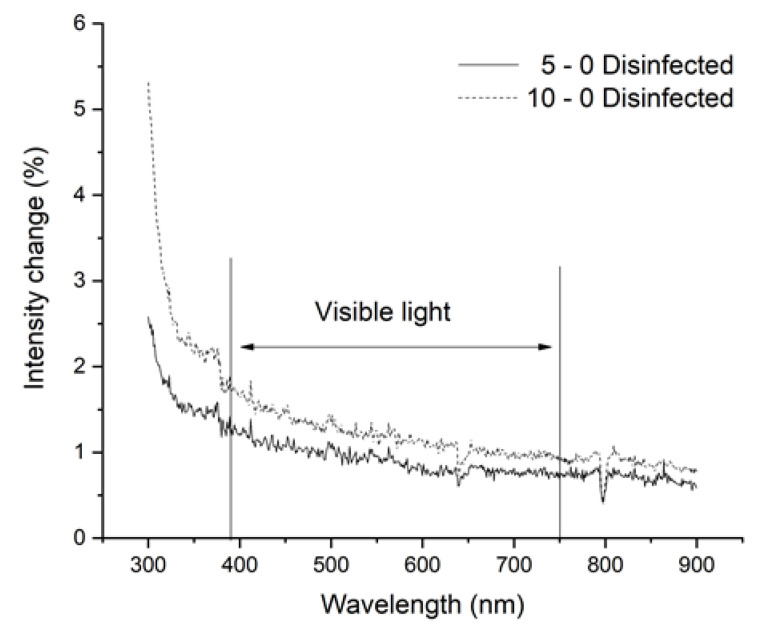
Results of spectrophotometry. The measurements were performed between 300 and 900 nm wavelength. The threshold of visible light is indicated. The changes in intensity have been visualized (%), where the smooth line marks the results of test samples after five cycles, and the dotted line represents the test samples after 10 cycles. The values of intensity are subtracted from the baseline, which was determined using non-disinfected samples.

In the case of face shields and goggles, visibility is a key factor; therefore, the light intensity passing through the PMMA sheets is essential to the proper use of these PPE. Medical personal and other healthcare staff must clearly see through them, so disinfection methods should not affect their vision. The spectrophotometry measurements revealed that five or even 10 disinfection cycles did not decrease the light intensity significantly; interestingly, a small amount of increase (1–2%) can be observed (Figure 6).

### 3.3. Possible Threats, Hazards, and Practical Aspects of OS Models

In general, FFF technology has some disadvantages and thus poses potential hazards in PPE production. First, the layers of thermoplastic polymers can be easily separated from each other. It could be extremely dangerous if, for example, a half mask is produced, and the resultant small holes cause air leakage, thereby increasing the risk of infection. If the post-processing techniques involve removing support materials and if the parameters are not carefully maintained, the remaining small fins and barbs could hurt and irritate the skin, which could provide an avenue for several infections and cause avoidable discomfort. The majority of these problems can be solved using SLS 3D printing technology, where the integrity of the models is considerably higher than the models printed by FFF technology. The details of the findings and potential hazards regarding the OS models (Figure 1, Table 1) are summarized hereinafter:

- **OS Shield:** The initial design has three main disadvantages: it does not protect the top of the head, it has a relatively high printing time (100 min with FFF and 51 min with SLS/piece) and high material usage, and the shield part is not long enough to protect the whole face.
- **OS Half Mask:** The available open-source model has severe fitting problems if it is printed using PLA or other relatively rigid thermopolymers. A specific size filter with an unknown origin can be fitted inside, which reduces the compatibility with other products. In this form, the design has several air leakage possibilities around the filter holder. With certain design modifications, a more compact configuration could provide better safety features. Furthermore, using SLS technology would make it possible to reduce the material’s overall weight (one piece) to 59.09% and the cost to 78.73% of the original values.
- **OS Safety Goggles:** The initial model is promising, but the application with a half mask is fairly difficult. Safety goggles reduce the peripheral viewing angle, which could be dangerous in a clinical environment. Furthermore, adding a poly (methyl methacrylate) (PMMA) or polyethylene terephthalate glycol (PETG) sheet is not practical, which reduces the possibility of applying disinfection measures.

### 3.4. Potential Development Aspects

In this study, the OS models were modified using different CAD software solutions (Autodesk Inventor 2020™, San Rafael, USA; Rhino 6™ (Rhinoceros McNeel, Barcelona, Spain); Fusion 360™, Autodesk, San Rafael, USA). Based on the findings and practical aspects, the research team created models that are cost-effective, easy to print and assemble, and adaptable for special or unique needs, and they can potentially increase the effectiveness and protection level of each device. The novel models are referred to as V.2.0.

**Figure 7:**
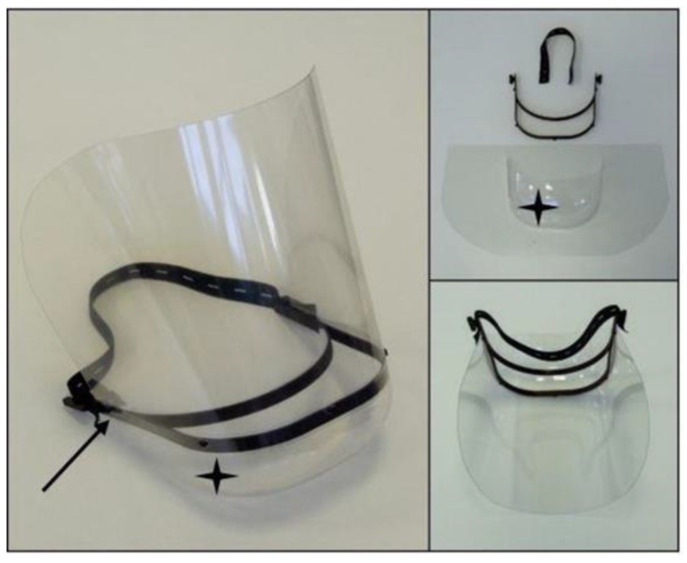
Shield V.2.0: The model improvements are marked as follows: Elongated head-mounted area to cover the face (black arrow) and adaptable cover for the top of the head (black star).

Shield V.2.0: The part of the model where the transparent polymer (PMMA) shield can be fitted is elongated to cover and protect a larger area of the face. The overall size of the model is decreased compared with the earlier model, which reduces the printing time in the case of FFF technology from 100 to 38 min for one piece. With SLS technology, the productivity is highly increased with the new model, as 41 pieces can be printed out in a full chamber. It was also important to design a cover to prevent contamination of the top of the head. This cover is fabricated from the same material used for the shield part (Figure 7). The material can withstand the standard disinfection process with tetra-acetyl-ethylene-diamine (TAED) (Sekusept™—EcoLab Hungary Ltd., Budapest, Hungary) without loss of transparency. The 3D-printed parts can also be reused, even after 10 cycles of disinfection.

**Figure 8:**
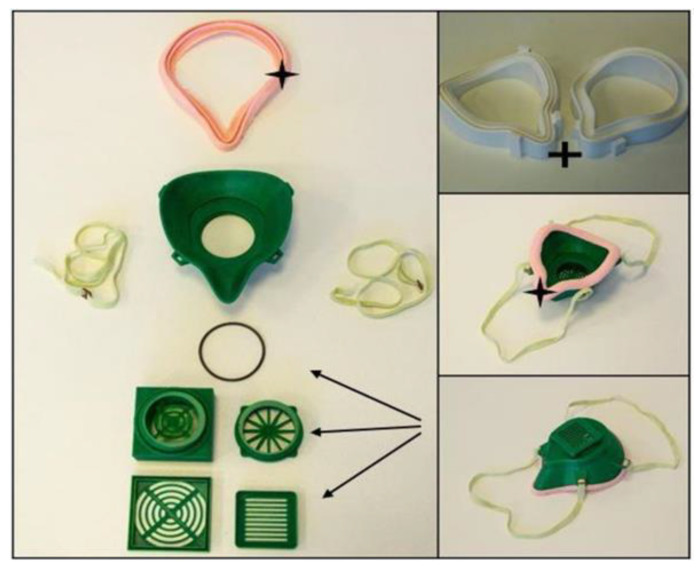
Face Mask V.2.0: The model improvements are marked as follows: interchangeable and modifiable filter holder parts with an O-ring (black arrows). Silicone ring layer for better face-fitting (black star). Mold for the silicone ring, printed from polylactic acid (PLA) with fused filament fabrication (FFF) technology (black cross).

Face Mask V.2.0: In general, half-face masks are extremely important in the prevention of COVID-19. The available OS models generally seek certifications to provide information about the level of protection (e.g., FFP or N levels). To receive a certification, one of the major criteria is fitting to the face and nose, since a good fit ensures that virus particles cannot enter the airway system. To improve the face fitting in the V.2.0 model, a silicone layer was designed to reduce air leakage, which could be easily molded by an FFF 3D-printed mold tool. As the flex resistance test revealed, the used silicone is a durable and long-lasting solution, which can be disinfected without affecting the mechanical stability. Since a proper and easily changeable filter holder is a crucial safety feature of the mask, a solution for this part was also established. To further prevent air leakage and increase safety, an O-ring can be inserted into the connector part. The material weight for one piece is reduced from 120 to 70 g with FFF printing technology, and the price is reduced from 0.73 to 0.43 Euros. In the case of SLS models, a silicone layer can also be used (Figure 8).

**Figure 9:**
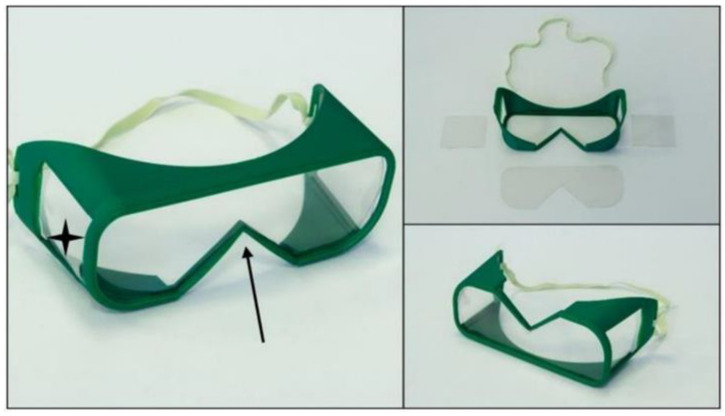
Safety Goggles V2.0. The model improvements are marked: new openings on the side of the model (black stars); the modified part for better fitting with face masks (black arrow).

Safety Goggles V2.0: Two main modifications have been applied for the new model. To provide a wider angle for peripheral view, two side openings were designed in the goggles, which can be covered with transparent polymer sheets. The changing of the transparent sheets is easy and fast, which helps in the disinfection process. The nose fitting was also redesigned to enable parallel use with a face mask. In terms of productivity, the use of SLS 3D printing technology is recommended (Figure 9). The spectrophotometry measurements confirmed that PMAA sheets can be a suitable solution, as the transparent part of the goggles and the disinfection cycles have no effect on the visibility or mechanical properties.

## 4. Discussion

AM technologies have had a serious impact on the development and production of PPE worldwide. Both FFF and SLS printers play a role in this field based on their characteristics and available printable materials. PLA is a suitable option for face shield production due to the cost-effectiveness and wide availability of the devices. In the case of supply shortages, as occurs in the COVID-19 pandemic, FFF technology can provide a temporary solution for PPE production, especially with previously described methods for strengthening functional 3D-printed parts [63]. FFF printing is a useful tool in half mask development and prototype printing; however, it is not recommended for final production due to its mechanical and structural characteristics. Safety goggles can be printed with both FFF and SLS, without any special hazards or risks. In terms of productivity, SLS technology with PA material is considerably more efficient than FFF technology for PPE production. It is important to note that the availability of SLS technology has increased since the cost-effective, desktop models appeared on the market [64]. Due to the recommended prudent use of PPE devices, it is extremely important to use proper disinfection protocols in the case of 3D-printed devices according to the surface characteristics to reduce the chance of survival and transmission of SARS-CoV-2 [33,35,58]. Furthermore, recent scientific works propose carefully choosing the right 3D printing materials and printer nozzle [65]. Neither PLA nor PA should be sterilized with heat-based solutions such as steam, autoclaves, or dry heat methods, although PA can withstand a few cycles of these processes [66,67]. Ethylene oxide and alcohol-based disinfectants can potentially be used for disinfection in the case of both materials as cost-effective, widely available, common disinfection methods [66,68,69,70]. Gamma radiation can also be used, but only with special care [66,68]. Based on the available scientific results, heat-based disinfection methods and alcohol-based disinfectants are possibly effective against SARS-CoV-2, but further investigations are necessary [40,42,52]. Our study has revealed that the broadly available, cost-effective solution of tetra acetyl ethylene diamine (TAED) and sodium perborate solution (Sekusept™—EcoLab Hungary Ltd., Budapest, Hungary), which is an effective disinfectant against SARS-CoV-2, can be used as a sanitizer on polyamide, PLA, and ZA-22 silicone materials, without affecting the mechanical and structural integrity of the 3D-printed and molded parts [45]. It can also be concluded that disinfection of the transparent PMMA sheets did not reduce the transparency. The observations indicate that 3D-printed PPE are safely reusable several times when using the proper disinfection protocols.

In light of the enormous demand for PPE, companies and individuals have started to make 3D-printed masks, shields, and goggles. It is especially important to mention that the majority of these devices have not been approved by the Food and Drug Administration (FDA) or the European Community (CE); therefore, potential users must be aware of this fact and potential hazards. Some 3D-printed half masks have already gained preliminary approval from the FDA or obtained FFP3 equal validation (EN 140:1999 norm. CIIRC R95-3D), and they are meant for public use [48,71,72,73]. Further clinical trials on PPE created with AM technologies are mandatory [13,15,44,48].

## 5. Conclusions

In conclusion, during the COVID-19 pandemic, 3D printing technologies can help in preventive measures as an effective tool for PPE development and small-series production. Compared to other production methods such as injection molding, the lead time can be decreased, since there is no need for molding tool design. This fact also decreases the upfront cost. However, it is important to emphasize that after reaching the break-even point of production, 3D printing technologies are not sufficient from an economic perspective. According to previous studies, this break-even point can be estimated at around 200–300 product pieces, but the values strongly depend on the complexity and mass of the model [74,75]. FFF and SLS additive manufacturing technologies can serve as a reliable but temporary on-site or short-distance solution for PPE production in shortage of continuous supply. The presented and shared designs can potentially enhance the effectiveness of protection, and with further modifications, end products can be developed. Standard disinfection protocols could be used on the presented materials without affecting the usability, consequently offering a more environmentally friendly and rational re-use of the 3D-printed PPE. Companies or individuals must be aware of the appropriate material selection, as well as the local legal aspects of medical device regulations. Healthcare personnel directly working with COVID-19 patients must use only validated and approved PPE with minimum N95 safety level [76].

## Figures and Tables

**Table 1:** Summary of production properties of PPE devices in case of fused filament fabrication (FFF) and selective laser sintering (SLS) 3D printing.

	Product Time (1 pc) (min)		Material Weight for 1 Piece (g)		Material Cost for 1 Piece (EUR)		Pieces with Full Capacity (pcs)		Production Time for Full Volume (min)		Material Weight for full Capacity (g)		Material Cost for Full Capacity (EUR)	
Model Name	FFF	SLS *	FFF	SLS	FFF	SLS **	FFF	SLS	FFF	SLS *	FFF	SLS	FFF	SLS **
OS Shield	100	51	47	43	0.29	6.49	2	18	200	919	94	1305	0.57	116.87
Shield V.2.0	38	27	18	17	0.11	1.06	2	41	75	1125	36	488	0.22	43.66
OS Half Mask	157	75	120	111	0.73	6.11	3	14	470	1046	360	956	2.20	85.59
Mask V.2.0	167	90	70	65	0.43	7.77	3	12	500	1083	210	1042	1.28	93.25
OS Safety Googles	80	54	30	28	0.18	2.25	4	20	320	1081	120	503	0.73	45.03
Safety Goggles V.2.0	100	56	43	40	0.26	3.12	3	16	300	896	129	557	0.79	49.85
Material cost estimation		PLA/kg:	EUR 22.04		PLA/kg:	EUR 89.53		EUR/HUF:		363				

* Heating and cooling time is not included. ** Unused material price is not calculated.

## Supplementary Materials

The raw data required to reproduce these findings are available to download from https://data.mendeley.com/datasets/pvs6hfhpph/draft?a=44bc7506-88c1-4079-a3ce-7bfdfc54b365; Maroti, Peter; Pentek, Attila; Toth, Luca (2020), “3D Printed Personal Protective Equipments”, Mendeley Data, v1. The “.stl” format models are the following: Open Source Models library: Mask, Safety goggle, Shield. V2.0 Models: Mask, Safety goggles, Shield; http://dx.doi.org/10.17632/pvs6hfhpph.1.
